# Preparation and Performance Study of Three-Layer Composite Filter Media for Channel-Type Ultra-Low Penetration Air Filters

**DOI:** 10.3390/nano16100607

**Published:** 2026-05-15

**Authors:** Mingyu Li, Desheng Wang, Yuhan Wang, Jinhao Xie, Yuqiu Liu, Yun Liang, Jian Kang, Hao Wang

**Affiliations:** 1 School of Light Industry and Engineering, South China University of Technology, Guangzhou 510641, China; limingyu200128@163.com (M.L.); wyh8927@163.com (Y.W.); 13396429794@163.com (Y.L.); 2 State Key Laboratory of Chemistry for NBC Hazards Protection, Beijing 102205, China; deshen1982@163.com (D.W.); 15530958615@163.com (J.X.)

**Keywords:** channel-type filter, ultra-low penetration air filtration, three-layer composite filter medium, glass wool fiber, pressure drop

## Abstract

To satisfy the requirements of channel-type ultra-low penetration air (ULPA) filters for high filtration efficiency, low pressure drop, and good corrugation processability, a three-layer composite filter medium with a bast-fiber surface layer/glass wool–lyocell blended core layer/bast-fiber surface layer structure was designed and prepared. The effects of surface-layer material, core-layer fiber composition, surface-layer basis weight, and processing conditions on the overall performance of the medium were systematically investigated. Bast-fiber paper exhibited the best corrugation processability and mechanical performance and was selected as the surface layer. The optimal core-layer composition was 25 wt.% 475-79 glass wool fibers, 30 wt.% 475-59 glass wool fibers, and 45 wt.% lyocell fibers, yielding an original-sheet filtration efficiency of 99.9996% and a pressure drop of 381 Pa. Further optimization showed that a bast-fiber surface layer with a basis weight of 15 g/m^2^ provided the best balance among pleat retention, structural stability, and low-resistance characteristics. Under optimized corrugation conditions of 120 °C roller temperature, 10 m/min roller speed, and 0.480 mm roller gap, a desirable pleat morphology suitable for channel-type structures was obtained. The resulting channel-type ULPA filter maintained a filtration efficiency of 99.99954%, while increasing the effective filtration area by 51.6% and reducing the pressure drop by 26.1% compared with a conventional pleated filter with the same dimensions. These results provide a useful reference for the design and application of low-resistance, high-efficiency filter media for channel-type ULPA filters.

## 1. Introduction

In recent years, with the continuous advancement of industrialization and urbanization, energy consumption, industrial emissions, and transportation activities have increased significantly, leading to increasingly serious particulate air pollution, which has become an important factor affecting ecological security and public health [1]. Particulate matter, especially PM2.5 and smaller particles, is characterized by small particle size, large specific surface area, and long atmospheric residence time. These particles can penetrate deeply into the respiratory tract and deposit in the alveolar region, while also adsorbing toxic components such as heavy metals, organic pollutants, and biological contaminants, thereby posing more complex health risks [2,3,4,5,6,7]. In addition to outdoor air pollution, indoor particulate pollution should not be neglected. With the widespread use of highly airtight buildings and centralized air-conditioning systems, the development of efficient, low-resistance, and energy-saving air filtration technologies is of great significance for improving air quality and protecting public health [4].

Among various air purification technologies, fibrous filtration has been widely used in HVAC systems, cleanrooms, medical and healthcare environments, biosafety facilities, and electronic manufacturing because of its simple structure, high treatment efficiency, stable operation, and broad applicability. In particular, ultra-low penetration air (ULPA) filters are capable of extremely high removal efficiency for submicron particles and are key devices for achieving highly clean air environments [8,9]. Conventional pleated filters increase the effective filtration area per unit volume by pleating the filter medium. However, as the number of pleats increases and pleat spacing decreases, airflow channels become restricted, local flow-field distribution becomes uneven, and pressure drop rises, resulting in an inherent trade-off between filtration area enhancement and resistance increase [10]. Therefore, reducing pressure drop while maintaining ultra-high filtration efficiency has become an important direction in the development of high-performance air filters.

Channel-type filters form alternating sealed flow channels, forcing air to first flow along the channel direction and then pass transversely through the filter medium. This design enables a larger effective filtration area and a more rational flow-field distribution within a limited volume, offering the advantages of compact structure, high filtration-area utilization, and low resistance [11,12,13,14,15]. Since the commercialization of related patents in 1998, channel-type structures have shown considerable potential in engine intake and air purification applications [11]. Previous studies have shown that the shape of corrugated pleats is closely related to channel dimensions, effective filtration area, and flow resistance, and that rational pleat design is beneficial for optimizing the balance between filtration efficiency and pressure drop [14]. For regular corrugated media commonly used in channel-type structures, when the ratio of the arc length of the medium to the channel width falls within an optimal range, more favorable flow and structural characteristics can be obtained. In experimental evaluation, a pleat height-to-width ratio of approximately 0.395–0.425 can be regarded as the optimal pleat-shape range. This range reflects the geometric stability of the formed filter medium and also provides an important basis for material design and process optimization in channel-type structures.

However, channel-type structures impose higher requirements on filter media. The medium must not only exhibit ultra-high filtration efficiency, but also possess good flexibility, mechanical strength, and corrugation processability. Existing commercially available media suitable for channel-type processing are mainly plant-fiber-based. Although these materials exhibit good formability, their filtration efficiency generally only reaches the sub-HEPA level. In contrast, glass-fiber media have excellent particle capture capability but usually suffer from high brittleness, poor folding resistance, and a tendency to crack during processing, making them difficult to apply directly in channel-type ULPA filters [16].

Nanofibrous materials have attracted extensive attention in high-performance air filtration because of their small fiber diameter, large specific surface area, and fine, tunable pore structure [17,18]. Nanoscale fibrous networks can significantly increase the probability of contact between particles and fibers, thereby enhancing the capture of submicron and nanoscale particles and improving filtration performance [19]. For conventional fibrous filter media, filtration performance is strongly affected by fiber structure, pressure drop, quality factor, most penetrating particle size, and aerosol loading behavior. Comparative studies of glass-fiber, PTFE membrane, and electret filter media have shown that different fibrous media exhibit distinct filtration characteristics due to differences in filtration mechanisms and structural features. In particular, glass-fiber media generally provide high filtration efficiency but tend to show relatively high pressure drop, indicating the need for structural optimization to achieve low-resistance high-efficiency filtration [20].

Meanwhile, multiscale fiber networks containing nanoscale fiber components also provide new design strategies for the synergistic optimization of high efficiency and low pressure drop. In the present work, the glass wool fiber system used in this study contains fibers with diameters down to the nanoscale, which gives it great potential for efficient particle capture but also leads to pronounced brittleness, limited structural stability, and insufficient engineering formability [21,22,23]. Particularly during corrugation processing and channel-type assembly, media that rely solely on ultrafine or nanoscale fibers often struggle to simultaneously achieve high filtration efficiency, low pressure drop [24,25,26], and satisfactory mechanical performance. Therefore, developing multiscale fiber combinations and structural designs that preserve the efficient capture ability of nano-/submicron-scale fibers while improving structural integrity and channel-type processability has become critical to the engineering application of high-performance filter media.

In this context, multilayer composite filter media containing highly efficient capture components and supporting/reinforcing components are considered an effective route to simultaneously achieve filtration efficiency, structural stability, and engineering processability. Through the construction of multilayer structures with gradient pore size and functional partitioning, large particles can be pre-captured in the upstream layer, fine particles can be efficiently retained in the intermediate layer, and structural stability and processability can be improved by the outer supporting layers. Previous studies have shown that double-layer or triple-layer composite structures can, to some extent, simultaneously optimize filtration efficiency, pressure drop, and mechanical properties, while also enabling additional functions such as antibacterial behavior, moisture transport, and environmental adaptability [27,28,29,30,31,32,33,34,35]. Nevertheless, existing studies have mainly focused on planar filtration materials or single-factor structural optimization, and systematic investigations of ultra-high-efficiency composite filter media for channel-type air filters remain limited, especially with respect to the coordinated optimization of surface-layer material selection, core-layer fiber composition, surface-layer basis weight, and corrugation processing parameters.

Accordingly, in this study, a three-layer composite filter medium for channel-type ULPA filters was designed with a “bast-fiber surface layer/glass wool–lyocell blended core layer/bast-fiber surface layer” structure. The effects of plant-fiber type, fiber composition of the filtration core layer, surface-layer basis weight, and corrugation processing parameters on the overall performance of the filter medium were systematically investigated. A channel-type ULPA filter was further fabricated to verify its advantages in filtration efficiency and low-resistance performance. This work is expected to provide a theoretical basis and experimental reference for the design and engineering application of channel-type high-performance filter media containing nanoscale fiber components.

The novelty of this work lies in the process-oriented design of a three-layer composite filter medium specifically for channel-type ULPA filters. Instead of developing new fiber materials, this study integrates commercially available bast fibers, glass wool fibers, and lyocell fibers into a functional multilayer structure. The bast-fiber surface layers provide corrugation-forming ability and surface support, whereas the glass wool–lyocell blended core layer provides ultra-high filtration efficiency and improved structural integrity. This design differs from conventional single-layer glass-fiber ULPA media and general multilayer filter media because it simultaneously considers filtration performance, mechanical integrity, corrugation processability, and compatibility with channel-type filter assembly.

## 2. Materials and Methods

### 2.1. Raw Materials

The raw materials used in this study mainly included plant fibers for the surface layers, fibrous materials for the filtration core layer, and auxiliary materials for lamination. The plant fibers used for the surface layers included bast fibers, softwood fibers, and hardwood fibers, which differ in fiber length, flexibility, and network-forming ability. Bast fibers generally have relatively long and slender morphology, good flexibility, and strong inter-fiber entanglement ability, which are beneficial for forming a continuous supporting network and improving corrugation processability. Softwood fibers also possess a relatively long fibrous structure and can provide a certain degree of mechanical support, whereas hardwood fibers are usually shorter and finer, resulting in weaker skeletal support and lower deformation adaptability during corrugation processing. Bast fibers, softwood fibers, and hardwood fibers, all purchased from Guangxi Huaxian New Materials Co., Ltd. (Guangxi, China), were used as candidate raw materials for the plant-fiber surface layers to compare the corrugation processability of different plant-fiber papers and screen suitable surface-layer materials. The filtration core layer was composed of 475-79 glass wool fibers, 475-59 glass wool fibers (Yulin Tianshengyuan Glass Fiber Technology Co., Ltd., Yulin, China), and lyocell fibers (Hangzhou Youbiao Technology Co., Ltd., Hangzhou, China), aiming to construct a fibrous network with both high filtration efficiency and good processing performance. Among them, 475-79 and 475-59 glass wool fibers served as the main high-efficiency filtration components because their small fiber diameters help increase the specific surface area of the fibrous network and reduce the characteristic pore size, thereby enhancing particle capture. The 475-79 glass wool fibers were used to provide a basic fine-fiber framework, while the 475-59 glass wool fibers were adjusted together with lyocell fibers to regulate network density, filtration efficiency, and processability. Lyocell fibers, as cellulose-based flexible reinforcing fibers, were introduced to improve inter-fiber bonding, mechanical integrity, and corrugation stability of the core layer. During lamination, hot-melt nonwoven fabric (Nantong Ruibo Nonwoven Technology Co., Ltd., Nantong, China) was used as the interlayer bonding material, and thermosetting acrylic resin (Kunshan Sanwang Resin Co., Ltd., Suzhou, China) was used to reinforce the filter media.

### 2.2. Preparation of Plant-Fiber Surface Layers

Different plant fibers were weighed according to the designed amount, dispersed in water, and disintegrated using a fiber disintegrator. The resulting fiber slurry was transferred to a sheet former, diluted with additional water, and thoroughly stirred, after which plant-fiber papers were prepared by a wet-laid process. The obtained wet sheets were dried on a flat-panel dryer at 105 °C to obtain the base papers, which were then impregnated with thermosetting acrylic resin at an add-on level of 5 (±0.5)% to improve structural stability and mechanical strength.

Bast-fiber paper, softwood-fiber paper, and hardwood-fiber paper with a basis weight of 40 g/m^2^ were prepared to investigate the effect of fiber type on corrugation processability. In addition, bast-fiber papers with basis weights of 10, 15, 20, 30, and 40 g/m^2^ were prepared to study the effect of basis weight on medium performance.

### 2.3. Preparation of the Filtration Core Layer

The filtration core layer was prepared by a wet-laid process using 475-79 glass wool fibers, 475-59 glass wool fibers, and lyocell fibers. The mass fractions of the components are listed in Table 1. In the formulation design of the filtration core layer, the content of 475-79 glass wool fibers was fixed to provide a basic fine-fiber network for high-efficiency particle capture. The contents of 475-59 glass wool fibers and lyocell fibers were varied to regulate the balance between filtration efficiency and processing adaptability. A higher proportion of fine glass wool fibers was expected to increase fiber surface area and network density, thereby improving filtration efficiency, whereas excessive glass wool fibers could increase brittleness and reduce corrugation stability. Lyocell fibers were introduced as flexible reinforcing fibers to improve mechanical integrity and processability, but excessive lyocell content could reduce the relative amount of fine filtration fibers and weaken filtration efficiency. Therefore, the fiber ratios listed in Table 1 were designed to optimize the balance between filtration performance and corrugation processability. After disintegration, mixing, sheet formation, and drying, the base core-layer papers were obtained and then further reinforced with thermosetting acrylic resin. To optimize the core-layer composition, samples with different glass wool fiber/lyocell fiber ratios were designed and compared. Detailed preparation procedures are provided in Figure S1.

### 2.4. Preparation of Three-Layer Composite Filter Media

Three-layer composite filter media were fabricated using plant-fiber paper as the surface layers and the glass wool/lyocell blended medium as the core layer, with hot-melt nonwoven fabric serving as the interlayer bonding material. During lamination, the hot-melt nonwoven fabric was placed between the surface layer and the core layer and then heated on a flat-panel dryer. The hot-melt nonwoven fabric was selected because it can melt under the lamination temperature and act as a thermal bonding layer without substantially changing the main fibrous structure of the surface layer or core layer. When the heating temperature reached 140 °C, the hot-melt nonwoven fabric melted and achieved interlayer bonding, thereby bonding the plant-fiber surface layers and the glass wool–lyocell core layer together and improving the integrity of the three-layer composite medium. The schematic diagram of the three-layer composite structure is shown in Figure S2.

### 2.5. Corrugation Processing

Corrugation processing was performed using a channel-type pleating machine. The device consisted of upper and lower forming rollers equipped with heating units and a transmission system. Roller temperature was monitored and controlled in real time using temperature sensors. The filter medium was fed between the forming rollers under traction and was shaped into a corrugated structure under the combined effects of heat and mechanical stress. The entire forming process can be divided into three stages: thermal softening, mechanical bending and shaping, and cooling fixation.

To investigate the effects of processing parameters on formability, the influences of roller temperature, roller speed, and roller gap on the pleat height-to-width ratio were systematically studied. A schematic of the equipment is provided in Figure S3.

### 2.6. Fabrication of Channel-Type Filters

Channel-type filters were fabricated using the optimized three-layer composite filter medium. The composite medium was first processed into corrugated sheets, which were then assembled with flat sheets to form channel-type filter sheets. These sheets were subsequently cut and assembled into channel-type filter elements. To compare the performance of different filter structures, a conventional pleated filter with the same dimensions was also prepared as a control. Detailed fabrication procedures are given in Figure S4.

### 2.7. Morphological Characterization

The surface and cross-sectional morphologies of the plant-fiber papers, filtration core layers, and composite filter media were observed using scanning electron microscopy (SEM, Gemini SEM 300, ZEISS, Jena, Germany). Prior to testing, samples were sputter-coated with gold to improve conductivity. Morphological analysis was used to compare the fiber shape and network structure of different plant fibers, observe the fiber distribution in core layers with different compositions, and analyze structural integrity, fiber fracture, and interlayer delamination before and after corrugation processing.

### 2.8. Pore Size Measurement of Channel-Type Filter Paper

The pore size of the filter paper was characterized using a capillary flow porometer (PMI, Newtown Square, PA, USA) in accordance with ASTM F316 [36]. The capillary flow porometer operates based on the bubble point method. Briefly, the filter paper was first fully wetted with a wetting liquid, commonly Galwick. Clean and dry air was then applied to displace the wetting liquid from the pores of the filter paper. As the applied pressure increased, the liquid in the pore channels was gradually expelled. By recording the relationship between gas pressure and flow rate, the mean pore size and maximum pore size of the filter paper were obtained.

### 2.9. Evaluation of Corrugation Processability

To more directly evaluate corrugation processability, the pleat height-to-width ratio was introduced as a key metric. Both pleat height and pleat width were measured from the geometric dimensions of the formed samples. A larger pleat height-to-width ratio indicates a more upright pleat morphology and better formability. In addition, the overall processability of the filter medium was comprehensively evaluated by considering whether cracking, sticking to the rollers, difficult demolding, or non-uniform pleat formation occurred during processing. The wave-shaped pleated structure is shown in Figure S5.

### 2.10. Mechanical Property Testing

The tensile strength of the filter media was measured with reference to GB/T 12914-2018 [37], Paper and board—Determination of tensile properties—Constant rate of elongation method (20 mm/min). Before testing, the samples were conditioned under standard atmospheric conditions. The specimen width was 15 mm, the gauge length was 100 mm, and the tensile speed was 20 mm/min. By comparing the tensile strength and elongation of different plant-fiber papers, filtration core-layer formulations, and composite media with different surface-layer basis weights, the structural stability and damage resistance of the materials were evaluated.

### 2.11. Filtration Performance Testing

The filtration performance of the filter media was evaluated using the oil-mist method. No. 32 turbine oil was used to generate the oil-mist aerosol. The generated oil aerosol had a count median diameter of 0.185 μm and a mass median diameter of 0.33 μm. During the test, the upstream oil-mist concentration was maintained at 2500 mg/m^3^, and the face velocity for flat-sheet filter media testing was 5.33 cm/s.

Before data collection, the oil-mist aerosol generation system and the filtration test system were allowed to reach a stable operating state. The upstream and downstream oil-mist concentrations were then measured to determine the filtration efficiency of the filter media. The pressure drop across the filter medium was recorded simultaneously using a differential pressure measuring device. The testing equipment used in this study complied with GB/T 6165-2021 [38], Test Method of the Performance of High Efficiency Particulate Air Filter—Efficiency and Resistance. The test setup is shown in Figures S6 and S7.

## 3. Results and Discussion

### 3.1. Effect of Plant Fiber Type on the Corrugation Processability of Surface Layers

**Figure 1:**
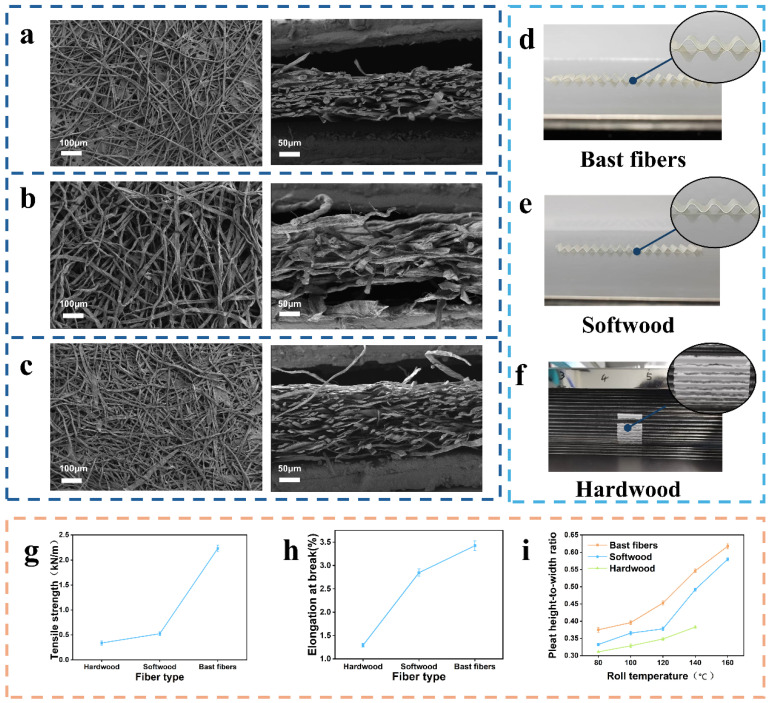
(**a**) Surface and cross-sectional SEM images of bast-fiber paper; (**b**) surface and cross-sectional SEM images of softwood-fiber paper; (**c**) surface and cross-sectional SEM images of hardwood-fiber paper; (**d**) images of corrugated bast-fiber paper; (**e**) images of corrugated softwood-fiber paper; (**f**) images of corrugated hardwood-fiber paper; (**g**) tensile strength of the three fiber papers; (**h**) elongation of the three plant-fiber papers; (**i**) pleat height-to-width ratio of the three plant-fiber papers at different roller temperatures.

To identify a suitable surface-layer material for channel-type ultra-high-efficiency composite filter media, bast-fiber paper, softwood-fiber paper, and hardwood-fiber paper were first compared in terms of microstructure, mechanical properties, and corrugation behavior. As shown in Figure 1a–c, the three plant-fiber papers exhibited distinct differences in fiber morphology and network structure. The bast-fiber paper consisted mainly of long, slender fibers intertwined into a continuous network, with a clearly layered stacked structure in the cross-section. Softwood-fiber paper retained typical tracheid morphology and hollow lumens, resulting in a relatively loose overall structure. Hardwood-fiber paper, in contrast, was mainly composed of shorter and finer fibers, with tighter inter-fiber bonding and a more uniform structure, but with limited skeletal support capability.

Under identical roller gap and roller speed conditions, the three plant-fiber papers were subjected to corrugation processing at roller temperatures of 80, 100, 120, 140, and 160 °C. The results showed clear differences in processability among the three materials, especially at 160 °C. As shown in Figure 1d, bast-fiber paper maintained a relatively intact structure and smooth surface throughout the investigated temperature range and exhibited good demolding stability even at high temperature. As shown in Figure 1e, softwood-fiber paper was able to form relatively upright pleats, but the processed surface became rough, with local fiber shedding and slight roller sticking. As shown in Figure 1f, hardwood-fiber paper exhibited poor stability at high temperature; at 160 °C, it fractured and adhered to the roller surface, making complete release from the rollers impossible. These results indicate that bast-fiber paper outperformed the other two plant-fiber papers in terms of structural integrity, pleat quality, and processing stability.

Mechanical testing further explained these differences. As shown in Figure 1g,h, the tensile strength and elongation of bast-fiber paper were 2.23 kN/m and 3.22%, respectively, both significantly higher than those of softwood-fiber paper (0.48 kN/m and 2.85%) and hardwood-fiber paper (0.32 kN/m and 1.29%). The higher tensile strength indicates stronger resistance to damage during thermo-mechanical bending, while the higher elongation enables the material to accommodate greater deformation without fracture, both of which favor the maintenance of structural integrity and high pleat morphology during corrugation. The superior tensile performance of the bast-fiber paper can be further attributed to the intrinsic characteristics of bast fibers. Bast fibers generally possess relatively long fiber length, good flexibility, and strong inter-fiber entanglement ability, which allow them to form a continuous and mechanically stable fiber network. This network structure improves the load-bearing capacity of the paper and helps dissipate stress during bending deformation. Although softwood fibers also have relatively long fibers and can provide certain mechanical support, their network structure was relatively loose under the present preparation conditions, resulting in lower tensile strength and less stable surface quality during corrugation. In contrast, hardwood fibers are usually shorter and form a less entangled network, leading to weaker mechanical integrity and poorer deformation adaptability.

As shown in Figure 1i, the pleat height-to-width ratios of all three plant-fiber papers increased as the roller temperature increased, indicating that a higher roller temperature facilitates corrugation shaping of plant-fiber papers. Bast-fiber paper consistently showed the highest pleat height-to-width ratio throughout the temperature range, increasing from 0.380 at 80 °C to 0.623 at 160 °C. The ratio for softwood-fiber paper increased from 0.335 at 80 °C to 0.583 at 160 °C. For hardwood-fiber paper, the ratio increased from 0.312 at 80 °C to 0.381 at 140 °C, but at 160 °C the sample cracked and adhered to the roller surface, making it impossible to obtain a valid pleat morphology. These results indicate that, under the present processing conditions, increasing roller temperature continuously improved the corrugation processability of bast-fiber paper and softwood-fiber paper, whereas hardwood-fiber paper showed poor adaptation to high temperature and markedly lower processing stability.

Mechanistically, roller temperature is an important factor affecting the corrugation processability of plant-fiber papers. At lower temperatures, the paper was insufficiently heated, and the overall material flexibility is limited, making it difficult to conform to the forming roller under external pressure; therefore, the pleat height-to-width ratio remains low. As the roller temperature increased, the paper is heated more thoroughly, its bending deformability was enhanced, and thermal pressing more effectively induces shape formation, resulting in a continuous increase in the pleat height-to-width ratio. For bast-fiber paper and softwood-fiber paper, their fibrous networks possess better continuity and a certain load-bearing capability, allowing them to maintain structural integrity and form more upright pleats even at high temperature.

From a mechanistic standpoint, bast-fiber paper has clear advantages in cellulose content, fiber morphology, and mechanical properties. On the one hand, bast fibers have a relatively high cellulose content, giving the fibers themselves good strength and toughness. On the other hand, their long and slender morphology enables the formation of a continuous supporting framework and increases inter-fiber contact area and network stability under compression. Therefore, bast-fiber paper can both withstand relatively large bending strains and effectively maintain pleat geometry during corrugation processing. By comparison, softwood-fiber paper has some supporting ability but a looser network, leading to poorer surface quality and processing stability, while hardwood-fiber paper, due to its shorter fibers and weaker spatial entanglement ability, is more prone to structural failure under high-temperature thermo-compression.

Based on the combined results of morphology, mechanical properties, and corrugation behavior, bast-fiber paper was selected as the surface-layer material for the three-layer composite filter medium.

### 3.2. Effect of Fiber Composition on the Performance of the Filtration Core Layer

To obtain a filtration core layer with both ultra-high filtration efficiency and good processability, 475-79 glass wool fibers, 475-59 glass wool fibers, and lyocell fibers were blended, and the morphology, filtration performance, mechanical properties, and corrugation processability of different formulations were systematically investigated. The 475-79 and 475-59 glass wool fibers mainly contributed to particle capture by forming a fine fibrous network with high specific surface area and small characteristic pore size. The combination of these two glass wool fibers helped construct a multiscale network, which increased the probability of particle–fiber contact and improved filtration efficiency. Lyocell fibers mainly acted as flexible reinforcing components in the core layer. Their cellulose-based fiber structure improved the mechanical integrity and processing adaptability of the medium, reducing brittleness and structural damage during corrugation. Therefore, the optimized glass wool–lyocell blend provided a balance between high-efficiency particle capture and corrugation processability.

**Figure 2:**
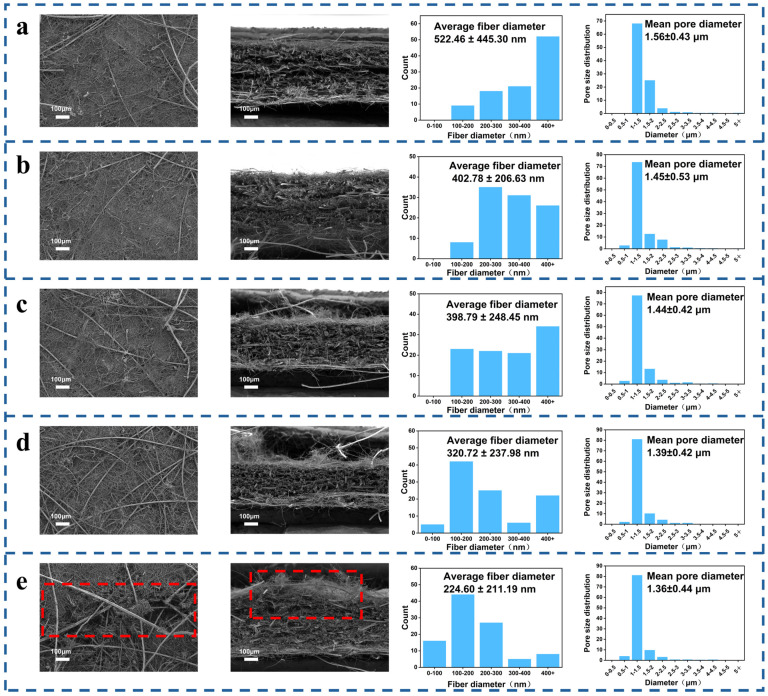
SEM images, fiber diameter distributions, and pore size distributions of filtration core layers with different fiber compositions: (**a**) 25% 475-79 glass wool fiber, 15% 475-59 glass wool fiber, and 60% Tencel fiber; (**b**) 25% 475-79 glass wool fiber, 20% 475-59 glass wool fiber, and 55% Tencel fiber; (**c**) 25% 475-79 glass wool fiber, 25% 475-59 glass wool fiber, and 50% Tencel fiber; (**d**) 25% 475-79 glass wool fiber, 30% 475-59 glass wool fiber, and 45% Tencel fiber; and (**e**) 25% 475-79 glass wool fiber, 35% 475-59 glass wool fiber, and 40% Tencel fiber. The red dashed boxes indicate the positions where fracture of the filtration material occurred.

As shown in Figure S8, all five core-layer formulations remained structurally intact after corrugation processing, without obvious visible cracks. The SEM images further reveal the multiscale fiber network of the filtration core layer. The fine glass wool fibers are distributed among the relatively coarser supporting fibers, forming a dense fiber network with reduced pore size. This morphology is beneficial for improving particle capture efficiency. Meanwhile, the lyocell fibers provide a flexible supporting framework and improve the structural continuity of the medium. The coexistence of fine glass wool fibers and lyocell fibers therefore contributes to both high filtration efficiency and improved processing stability. As shown in Figure 2, formulations 1–4 maintained complete structures after corrugation, with no obvious surface cracks, whereas formulation 5 exhibited slight surface cracking and fiber fracture in the cross-section, indicating that further increasing the glass fiber proportion significantly increased brittleness and reduced structural stability. Meanwhile, as the glass wool fiber content increased and the lyocell fiber content decreased, the proportion of fine fibers in the medium increased, and the average fiber diameter decreased from 522.46 nm to 224.60 nm, indicating that the fiber network gradually became denser.

The pore size distributions further confirm the evolution of the fibrous network structure with changing fiber composition. The average pore diameters of formulations 1–5 were 1.56 ± 0.43 μm, 1.45 ± 0.53 μm, 1.44 ± 0.42 μm, 1.39 ± 0.42 μm, and 1.36 ± 0.44 μm, respectively. This gradual decrease in average pore diameter indicates that increasing the proportion of fine glass wool fibers refined the pore structure and formed a denser multiscale fibrous network. The smaller characteristic pore size increased the probability of particle–fiber contact and shortened the transport distance required for particles to reach the fiber surface, which is favorable for improving filtration efficiency. From the perspective of filtration mechanisms, the nanoscale and submicron-scale glass wool fibers enhance Brownian diffusion capture for ultrafine particles, while the denser pore network strengthens interception for submicron particles. Therefore, the combined decrease in fiber diameter and pore size provides a structural basis for the ultra-high filtration efficiency of the glass wool–lyocell core layer.

**Figure 3:**
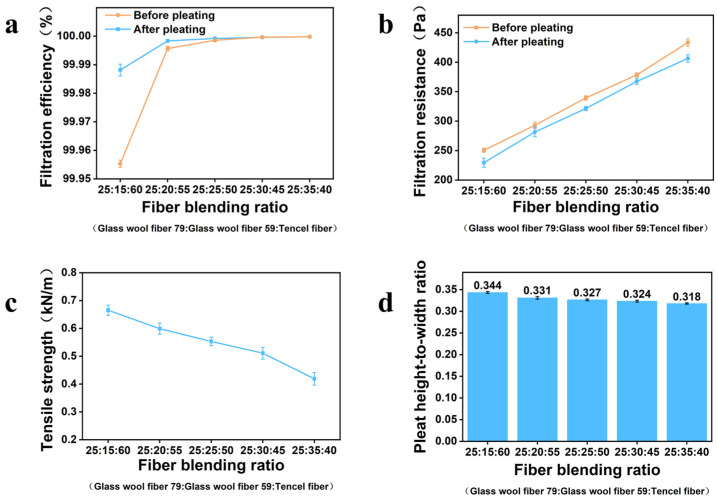
Performance comparison of filtration core layers with different fiber compositions: (**a**) filtration efficiency; (**b**) pressure drop; (**c**) tensile strength; (**d**) pleat height-to-width ratio.

In terms of filtration performance, the media with different formulations exhibited different trends before and after corrugation processing. As shown in Figure 3a, for samples with an initial filtration efficiency lower than 99.9995%, the filtration efficiency increased slightly after corrugation processing; for samples already above 99.9995%, the filtration efficiency remained essentially unchanged before and after processing. Meanwhile, as shown in Figure 3b, the pressure drop of all formulations decreased after corrugation processing, with a reduction of approximately 5%. The effect of corrugation processing on filtration performance can be mainly attributed to the change in the filtration structure and effective filtration area. Corrugation transformed the flat medium into a three-dimensional pleated structure, which increased the effective filtration area within the same projected test area and reduced the face velocity per unit filtration area. The reduced local face velocity lowered the airflow resistance through the porous medium, thereby decreasing the pressure drop after corrugation. For samples with relatively lower initial filtration efficiency, the increased effective filtration area and reduced local face velocity could slightly increase the probability of particle capture, leading to a slight increase in filtration efficiency after corrugation. However, for ultra-high-efficiency media whose initial filtration efficiency was already higher than 99.9995%, the original fibrous network was already sufficiently effective for particle capture. Therefore, the increase in effective filtration area was sufficient to reduce pressure drop to some extent but insufficient to further improve filtration efficiency significantly; consequently, the filtration efficiency remained essentially unchanged after corrugation. The filtration behavior of the core layer is mainly governed by Brownian diffusion, interception, and inertial impaction. The fine glass wool fibers provide a dense multiscale network with high specific surface area and small characteristic pore size, thereby increasing the probability of particle–fiber contact. Brownian diffusion contributes to the capture of ultrafine particles, while interception and inertial impaction become important for submicron and relatively larger particles. Lyocell fibers improve the continuity and flexibility of the fibrous network, helping the core layer preserve its filtration structure during corrugation. Thus, the optimized glass wool–lyocell blend achieves a balance between high-efficiency particle capture and corrugation processability.

As shown in Figure 3c, mechanically, decreasing the lyocell fiber content led to a continuous decrease in tensile strength. This is because lyocell fibers contain abundant hydroxyl groups on the surface and can readily form strong hydrogen bonds during wet-laid processing, whereas glass wool fibers primarily rely on physical entanglement to maintain network structure, resulting in weaker inter-fiber bonding. When the lyocell fraction decreases, the overall bonding strength of the fibrous network is reduced, leading to lower tensile strength.

As shown in Figure 3d, the corrugation processability results further showed that the pleat height-to-width ratio decreased slightly from 0.342 to 0.317 as the lyocell fiber content decreased. Lyocell fibers have good flexibility and elongation at break, and an appropriate amount of lyocell helps alleviate the brittleness of the glass-fiber network and improve crack resistance during bending deformation. By contrast, when the glass fiber fraction becomes too high, the medium becomes more susceptible to stress concentration at pleat peaks and valleys, resulting in cracking and structural failure.

Considering filtration efficiency, pressure drop, tensile strength, and pleat height-to-width ratio together, formulation 4 exhibited the best overall performance. Its composition was 25% 475-79 glass wool fibers, 30% 475-59 glass wool fibers, and 45% lyocell fibers. The original-sheet filtration efficiency was 99.9996%, and the pressure drop was 381 Pa, while maintaining good structural integrity and corrugation processability. Therefore, formulation 4 was selected as the filtration core layer for the subsequent three-layer composite filter media.

### 3.3. Effect of Surface-Layer Basis Weight on the Performance of Composite Filter Media

#### 3.3.1. Effect of Surface-Layer Basis Weight on Bast-Fiber Paper Performance

**Figure 4:**
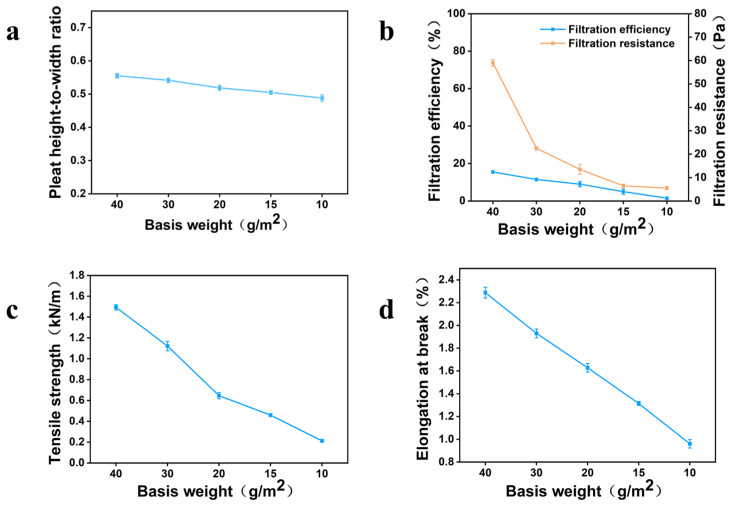
Performance comparison of bast-fiber papers with different basis weights: (**a**) pleat height-to-width ratio; (**b**) filtration performance; (**c**) tensile strength; (**d**) elongation.

After bast fiber was selected as the surface-layer raw material, the effects of basis weight on corrugation processability, filtration performance, and mechanical properties were further investigated. As shown in Figure 4a, as the basis weight of bast-fiber paper decreased, the pleat height-to-width ratio decreased from 0.550 to 0.495, but the overall decrease was relatively small, indicating that bast-fiber paper maintains good formability over a relatively broad basis-weight range. Even at 10 g/m^2^, the medium could still form relatively upright pleats, demonstrating that bast fibers themselves provide strong structural support capability.

As shown in Figure 4b, with decreasing basis weight, both filtration efficiency and pressure drop decreased. The filtration efficiencies of all bast-fiber papers were below 20%, and because the surface layer of the composite medium mainly serves supporting, protective, and formability-improving functions, its filtration efficiency was not the primary focus. In terms of resistance, when the basis weight was below 20 g/m^2^, the pressure drop was below 10 Pa, which is negligible compared with that of ultra-high-efficiency media.

On the other hand, as shown in Figure 4c,d, decreasing the basis weight also caused the tensile strength and elongation of bast-fiber paper to decrease simultaneously. This indicates that although a lower basis weight is beneficial for reducing resistance, it may weaken the protective effect of the surface layer on the core layer and reduce the structural stability of the composite. Therefore, the selection of surface-layer basis weight should not be based solely on resistance, but should comprehensively consider the corrugation processability, mechanical properties, and filtration performance of the resulting composite medium. Based on these results, the subsequent study focused on surface-layer basis weights of 10, 15, and 20 g/m^2^.

#### 3.3.2. Effect of Surface-Layer Basis Weight on the Performance of Three-Layer Composite Media

**Figure 5:**
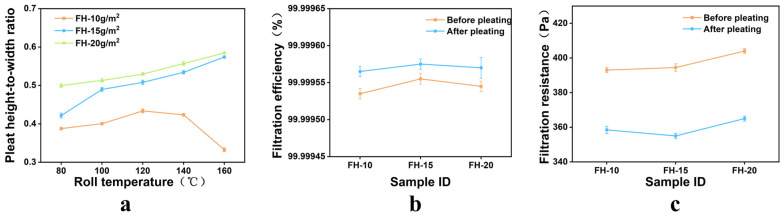
Performance of composite filter media with different surface-layer basis weights: (**a**) pleat height-to-width ratio; (**b**) filtration efficiency; (**c**) pressure drop.

As shown in Figure 5a, for the composite medium with a surface-layer basis weight of 10 g/m^2^, the pleat height-to-width ratio gradually increased with increasing roller temperature when the temperature was below 140 °C, reaching a maximum of 0.376 at 120 °C. When the roller temperature increased to 160 °C, the pleat height-to-width ratio decreased sharply. In contrast, for the composite media with surface-layer basis weights of 15 and 20 g/m^2^, the pleat height-to-width ratio increased continuously with increasing temperature and reached maximum values of 0.576 and 0.586, respectively, at 160 °C.

When the roller temperature exceeded 140 °C, the hot-melt nonwoven fabric reached its melting temperature and underwent remelting. For the 10 g/m^2^ sample, the low basis weight and thin surface layer resulted in incomplete coverage of the composite medium, exposing part of the interfacial region. The molten hot-melt nonwoven fabric came into direct contact with the roller surface and adhered to it, preventing smooth release of the medium and requiring forced removal. Because the pleat structure had not yet fully cooled and fixed during this stage, the tensile force applied during forced release caused a decrease in the pleat height-to-width ratio and a deterioration in pleat uniformity, as shown in Figure S9.

For the composite media with surface-layer basis weights of 15 and 20 g/m^2^, the thicker surface layer provided more complete coverage of both the core layer and the interlayer bonding material, resulting in better structural integrity and a lower risk of interfacial exposure and roller sticking under high-temperature conditions. As the roller temperature increased, the plant-fiber surface layer and the internal fibrous network of the composite absorbed heat more sufficiently and underwent more complete plasticization, allowing the medium to conform more readily to the roller surface and to be more fully shaped. Consequently, the pleat height-to-width ratio increased continuously and reached its maximum at 160 °C. This indicates that for composite media with relatively high surface-layer basis weights, increasing the temperature enhances formability rather than causing high-temperature instability due to insufficient coverage, as observed for the low-basis-weight sample.

From the standpoint of filtration performance, as shown in Figure 5b,c, after corrugation processing, all samples with different surface-layer basis weights exhibited slight increases in filtration efficiency while maintaining values above 99.9995%, and all exhibited decreases in pressure drop of 8.9–10.6%. These results indicate that the plant-fiber surface layers in the three-layer structure effectively protected the glass-fiber core layer during processing, preventing cracking induced by bending stress and thereby preserving the ultra-high filtration performance. At the same time, the corrugated structure increased the effective filtration area and reduced local face velocity, thus lowering the overall pressure drop.

In summary, when the surface-layer basis weight was 20 g/m^2^, the three-layer composite medium exhibited better corrugation processability and mechanical performance than the medium with a 15 g/m^2^ surface layer. However, considering that the optimal pleat height-to-width ratio range is 0.395–0.425, the composite medium with a 15 g/m^2^ surface layer satisfied this criterion. Therefore, a bast-fiber surface layer with a basis weight of 15 g/m^2^ was ultimately selected for the three-layer composite medium.

### 3.4. Effect of Processing Conditions on Corrugation Processability

After the composite structure was determined, the effects of roller temperature, roller speed, and roller gap on corrugation processability were further investigated.

**Figure 6:**
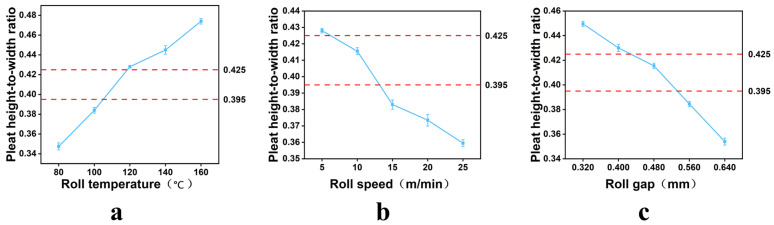
Effect of different processing parameters on the pleat height-to-width ratio: (**a**) roller temperature; (**b**) roller speed; (**c**) roller gap.

As shown in Figure 6a, under constant roller gap and roller speed, the pleat height-to-width ratio of the three-layer composite medium increased gradually from 0.351 to 0.473 as the roller temperature increased from 80 °C to 160 °C. At lower temperatures, the plant-fiber surface layer and the interlayer hot-melt bonding layer were insufficiently heated, and the material exhibited limited flexibility, making it difficult for the composite medium to fully conform to the roller surface; therefore, the resulting pleat morphology was relatively flat and the pleat height-to-width ratio was low. As the roller temperature increased, the plant-fiber surface layer gradually softened and the interlayer hot-melt nonwoven more easily reached its melting temperature, thereby enhancing the coordinated deformation capability of the layers and allowing the medium to bend more fully and form more upright pleats. Considering that the target pleat height-to-width ratio range for the channel-type structure was 0.395–0.425, the value of 0.427 obtained at 120 °C was closest to this range, and 120 °C was therefore selected as the optimal roller temperature.

As shown in Figure 6b, under constant roller temperature and roller gap, the pleat height-to-width ratio decreased gradually with increasing roller speed. At low roller speed, the medium stayed longer in the heated forming zone and could absorb heat more fully and undergo sufficient plasticization, thus forming higher pleats more easily. As roller speed increased, the heating and deformation time decreased, plasticization was insufficient, and elastic rebound after bending increased, resulting in a lower pleat height-to-width ratio. Although the highest ratio was obtained at 5 m/min, the value at 10 m/min was 0.417, which was closer to the target range; therefore, 10 m/min was selected as the optimal roller speed.

As shown in Figure 6c, under constant roller temperature and speed, the pleat height-to-width ratio decreased continuously as the roller gap increased. At smaller roller gap, the forming rollers applied stronger compression to the medium, and the fibrous network experienced greater stress during bending, which favored the formation of upright and stable pleats. As the roller gap increased, the compression decreased, the medium rebounded more easily, and the formed pleats became flatter. When the roller gap was 0.480 mm, the pleat height-to-width ratio was 0.417, which was closest to the target range, and this value was therefore selected as the optimal roller gap.

Based on the combined effects of these three factors, the optimal corrugation processing conditions for the three-layer composite medium were determined to be a roller temperature of 120 °C, a roller speed of 10 m/min, and a roller gap of 0.480 mm. Under these conditions, the resulting pleat height-to-width ratio was within the optimal range for the channel-type structure.

### 3.5. Performance of the Channel-Type ULPA Filter

**Figure 7:**
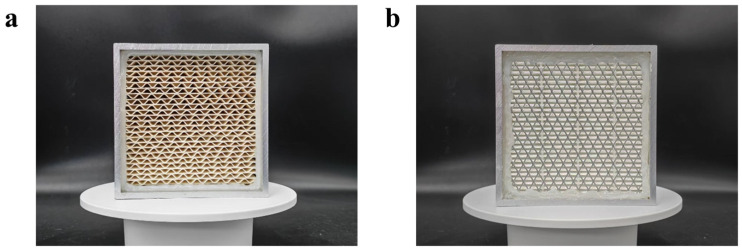
Photographs of (**a**) the channel-type filter and (**b**) the conventional pleated filter.

As shown in Figure 7a, a channel-type filter was fabricated under the optimized processing conditions. Figure 7b shows a conventional pleated filter of the same dimensions prepared from a commercial ULPA filter medium, which was used as a control to verify the structural advantages of the channel-type filter. The conventional pleated filter was selected for comparison because pleated structures are widely used in ULPA filters and represent a typical filter configuration in practical applications. By comparing the channel-type filter with a conventional pleated filter under the same external dimensions and test airflow rate, the influence of filter structure on effective filtration area, face velocity, and pressure drop could be evaluated more directly. Therefore, this comparison was mainly intended to clarify whether the channel-type structure could reduce flow resistance while maintaining ULPA-level filtration efficiency. The commercial filter medium used for comparison was an ultra-high-efficiency glass-fiber filter medium, with a basis weight of 100 g/m^2^, a filtration efficiency of 99.99956%, and an initial pressure drop of 375.4 Pa.

As shown in Table 2, at the same filter dimensions and test airflow rate (33 m^3^/h), the effective filtration area of the channel-type ULPA filter was 2616 cm^2^, which was 51.6% higher than that of the pleated filter (1725 cm^2^). Accordingly, the face velocity of the channel-type filter medium was reduced to 3.52 cm/s, compared with 5.33 cm/s for the pleated filter, representing a decrease of approximately 33.9%. On this basis, the pressure drop of the channel-type filter was 275.4 Pa, which was markedly lower than that of the pleated filter (372.6 Pa), corresponding to a reduction of 26.1%. The larger effective filtration area of the channel-type structure reduced the gas flux per unit area and thus lowered the flow velocity through the medium. According to the basic flow behavior of porous media, pressure drop typically increases with flow velocity; therefore, the reduction in face velocity directly reduces pressure drop. In addition, the straight-through flow characteristic of the channel-type structure can reduce local flow disturbance and energy loss within the pore structure, further lowering the pressure drop. Overall, by increasing the filtration area and reducing the face velocity, the channel-type ULPA filter effectively optimized the airflow distribution and reduced resistance, thereby extending service life, lowering energy consumption, and decreasing filter replacement frequency.

## 4. Conclusions

In this study, a three-layer composite filter medium with a “bast-fiber surface layer/glass wool–lyocell blended core layer/bast-fiber surface layer” structure was constructed, and the effects of surface-layer material, filtration core-layer fiber composition, surface-layer basis weight, and corrugation processing parameters on its overall performance were systematically investigated. The results showed that bast-fiber paper, owing to its excellent corrugation processability and favorable mechanical properties, was suitable as the surface layer of channel-type composite filter media. For the filtration core layer, when the mass fractions of 475-79 glass wool fibers, 475-59 glass wool fibers, and lyocell fibers were 25%, 30%, and 45%, respectively, the medium exhibited a desirable combination of filtration performance and structural integrity, with an original-sheet filtration efficiency of 99.9996% and a pressure drop of 381 Pa. The surface-layer basis weight further affected the forming stability and low-resistance characteristics of the composite medium, and a bast-fiber surface layer with a basis weight of 15 g/m^2^ achieved the best balance among pleat retention, structural stability, and pressure-drop control. Process optimization showed that increasing roller temperature favored a higher pleat height-to-width ratio, whereas increasing roller speed and roller gap weakened the forming effect; the optimal processing conditions for the channel-type structure were therefore determined to be a roller temperature of 120 °C, a roller speed of 10 m/min, and a roller gap of 0.480 mm. Based on these optimized conditions, the resulting channel-type ULPA filter maintained a filtration efficiency of 99.99954%, while increasing the effective filtration area by 51.6% and reducing the pressure drop by 26.1% compared with a conventional pleated filter of the same size. These results demonstrate that the proposed three-layer composite filter medium can effectively reconcile ultra-high filtration performance, structural stability, and channel-type formability and provides a reference for the design and application of low-resistance, high-efficiency filter media for channel-type ULPA filters.

Although the developed three-layer composite filter medium and the channel-type ULPA filter exhibited promising initial filtration performance and corrugation processability, several limitations of the present study should be acknowledged. First, the optimization of the bast-fiber surface-layer basis weight was mainly based on the pleat height-to-width ratio, because the surface layer primarily served as a corrugation-forming and surface-supporting layer in this work. However, additional properties related to long-term service, such as interlayer adhesion, delamination resistance, dust holding capacity, and pressure drop evolution during particle loading, were not quantitatively evaluated. Second, the present study mainly focused on the preparation, processing adaptability, and initial filtration performance of the fine filtration medium and channel-type filter. In practical applications, high-efficiency filters are generally used together with coarse filters in a two-stage filtration system, where the coarse filter reduces the particle loading burden of the fine filtration unit. Nevertheless, long-term performance indicators, including dust holding capacity, filtration efficiency stability, vibration resistance, service lifetime, and potential fiber shedding, remain important for engineering applications and require further investigation. Third, this study mainly analyzed the filtration mechanism from the perspectives of fiber morphology, fiber diameter, pore structure, network density, and mechanical integrity. Detailed surface chemical characterization, such as FTIR, Raman spectroscopy, and XPS analysis, was not included in the current experimental design. This is because the filtration performance of the present fibrous filter medium is mainly governed by physical structural factors, including fiber diameter, pore size, network density, and structural stability, rather than by chemical adsorption or surface functional groups. Nevertheless, surface chemistry and interfacial interactions may still influence fiber bonding, structural stability, and long-term performance. Therefore, future work may combine FTIR, Raman spectroscopy, or XPS analysis to further clarify the surface properties of the fibers and the interfacial interactions within the composite filter medium. Fourth, the scalability, production cost, and environmental impact of the multi-step preparation process, including wet-laid formation, resin impregnation, hot-melt lamination, and corrugation, were not assessed in detail. Future work will focus on long-term loading tests, structural durability evaluation, fiber shedding assessment, pilot-scale production, process simplification, alternative bonding methods, and recyclability analysis to further verify the practical applicability of the proposed composite filter medium and channel-type ULPA filter.

## Figures and Tables

**Table 1:** Fiber composition of the filtration core layer.

Formulation	475-79	475-59	Lyocell Fiber
1	25%	15%	60%
2	25%	20%	55%
3	25%	25%	50%
4	25%	30%	45%
5	25%	35%	40%

**Table 2:** Performance comparison between the channel-type ULPA filter and the pleated ULPA filter.

Filter Type	Filter Size (mm)	Filtration Area (cm^2^)	Filtration Efficiency (%)	Pressure Drop (Pa)
Channel-type ULPA filter	100 × 100 × 50	2616	99.99954	275.4
Pleated ULPA filter	100 × 100 × 50	1725	99.99953	372.6

## Data Availability

The original contributions presented in this study are included in the article/Supplementary Material. Further inquiries can be directed to the corresponding authors.

## Supplementary Materials

The following supporting information can be downloaded at https://www.mdpi.com/article/10.3390/nano16100607/s1: Figure S1. Preparation process flowchart of filter core layer material. Figure S2. Structure of three-layer composite filter media. Figure S3. Diagram of waveform pleating machine. Figure S4. Preparation flowchart of the channel-type filter. Figure S5. Structure diagram of waveform pleat. Figure S6. Principle diagram of filtration efficiency test system based on mass concentration. Figure S7. Schematic diagram of oil mist filter performance test device. Figure S8. Photographs of corrugated-pleat-processed glass wool fiber/Tencel fiber blends with different blending ratios. Figure S9. Photographs of composite filter media with three basis weight surface layers after pleating processing at different temperatures.
