# Supplementary material for: Preparation and Performance Study of Three-Layer Composite Filter Media for Channel-Type Ultra-Low Penetration Air Filters
Source: Nanomaterials (Basel). 2026 May 15;16(10):607. doi: 10.3390/nano16100607 (PMC13209818; doi:10.3390/nano16100607)
Supplement: Supplementary file 1 [file nanomaterials-16-00607-s001.zip › nanomaterials-4296865-supplementary.pdf]

Supplementary Material

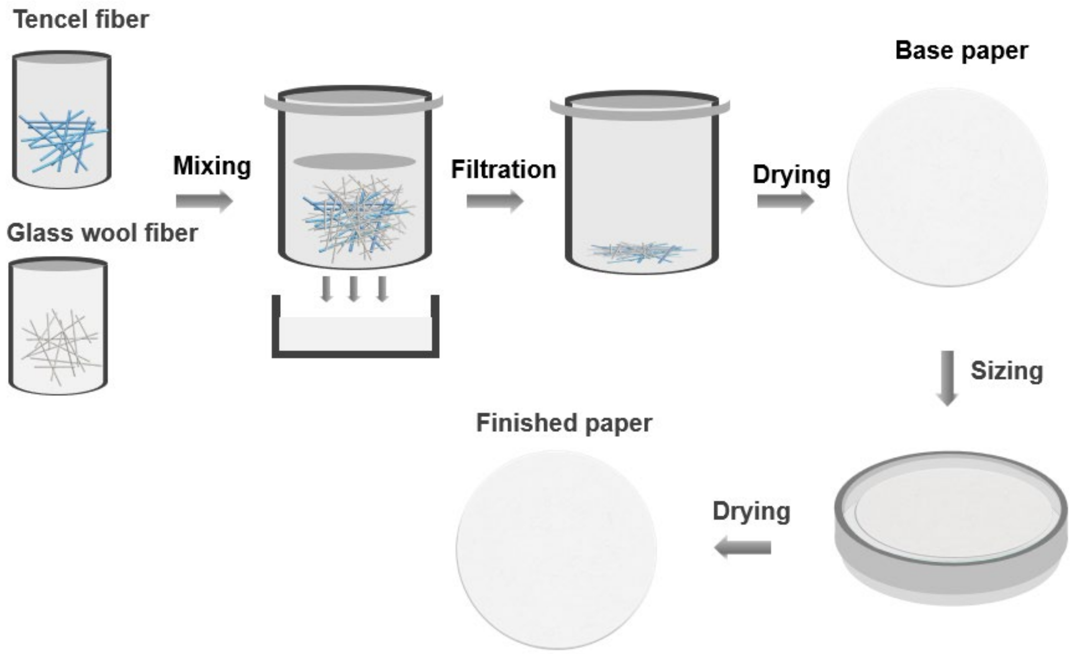

Figure S1. Preparation process flowchart of filter core layer material.

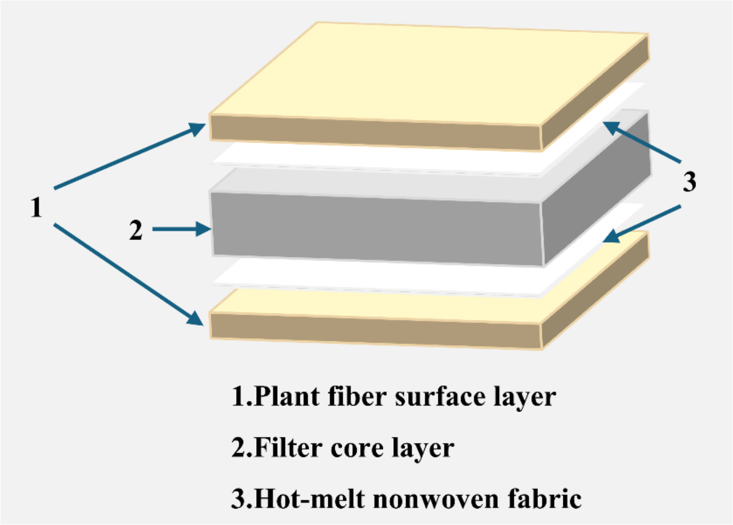

Figure S2. Structure of three-layer composite filter media.

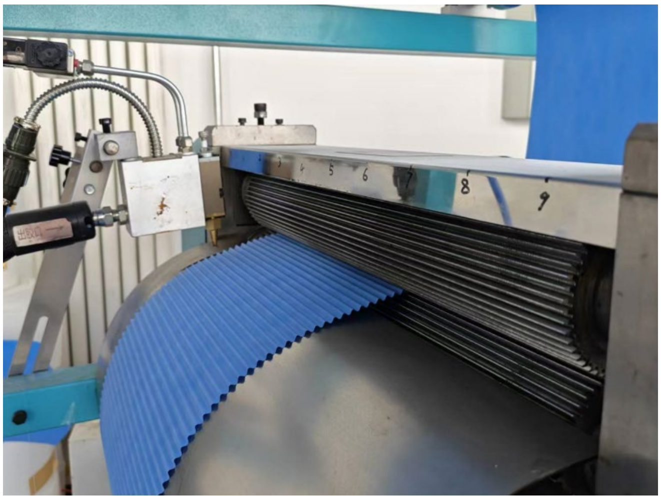

Figure S3. Diagram of waveform pleating machine.

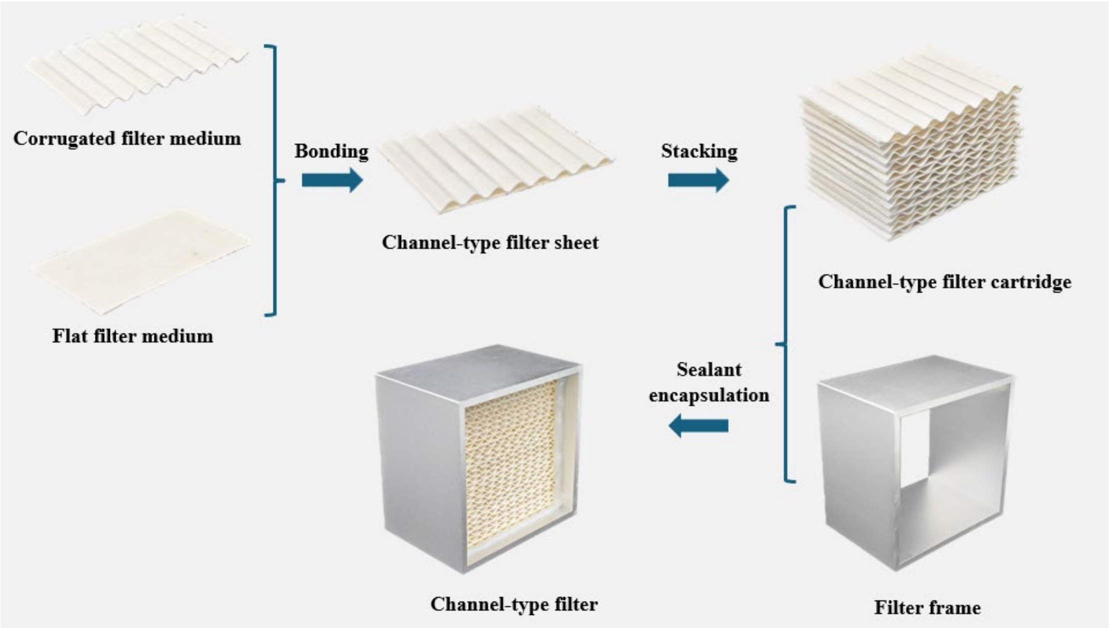

Figure S4. Preparation flowchart of the channel-type filter.

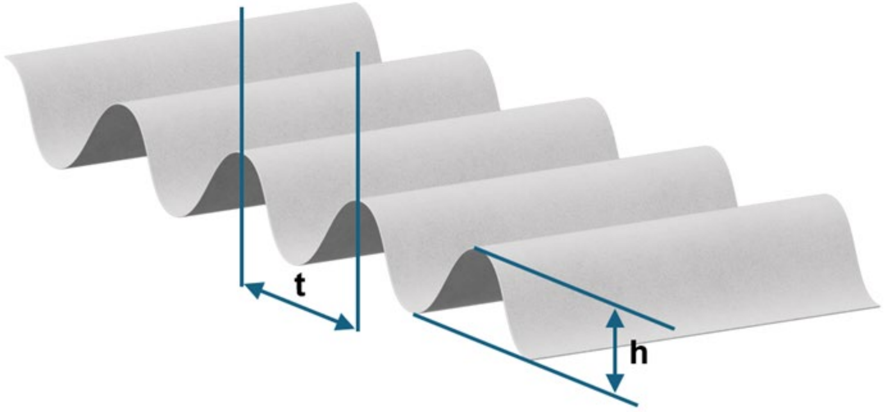

Figure S5. Structure diagram of waveform pleat.

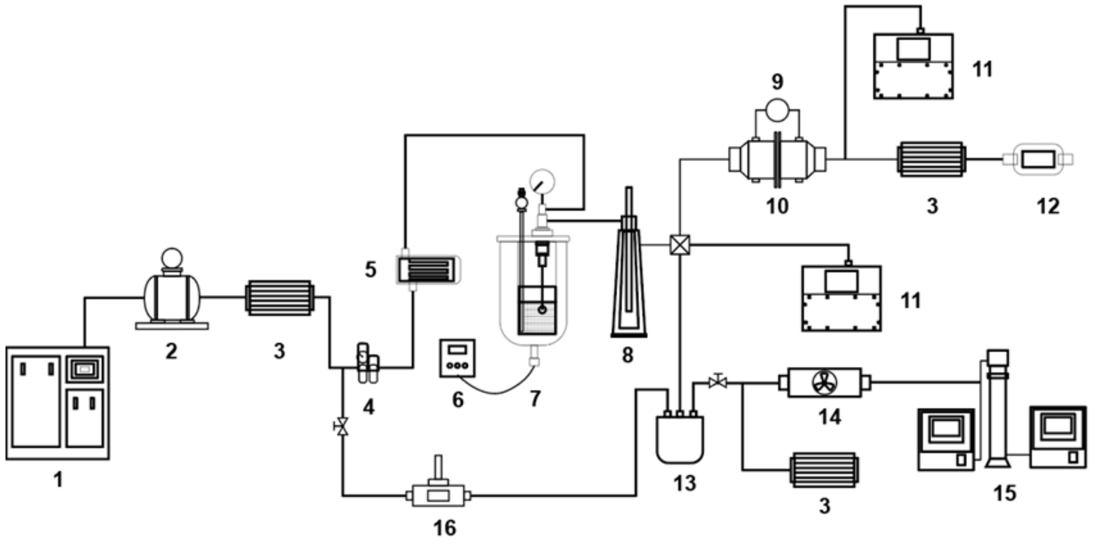

Figure S6. Principle diagram of filtration efficiency test system based on mass concentration. 1 - Air compressor; 2 - Pressure stabilizing tank; 3 - High-efficiency air filter; 4 - Pneumatic single unit; 5 - Air heating device; 6 - Temperature sensor controller; 7 - Oil mist generator; 8 - Spiral separator; 9 - Differential pressure gauge; 10 - Filter holder; 11 - Photometer; 12 - Flow meter; 13 - Mixing tank; 14 - Electrostatic neutralizer; 15 - Scanning mobility particle sizer; 16 - Flow controller.

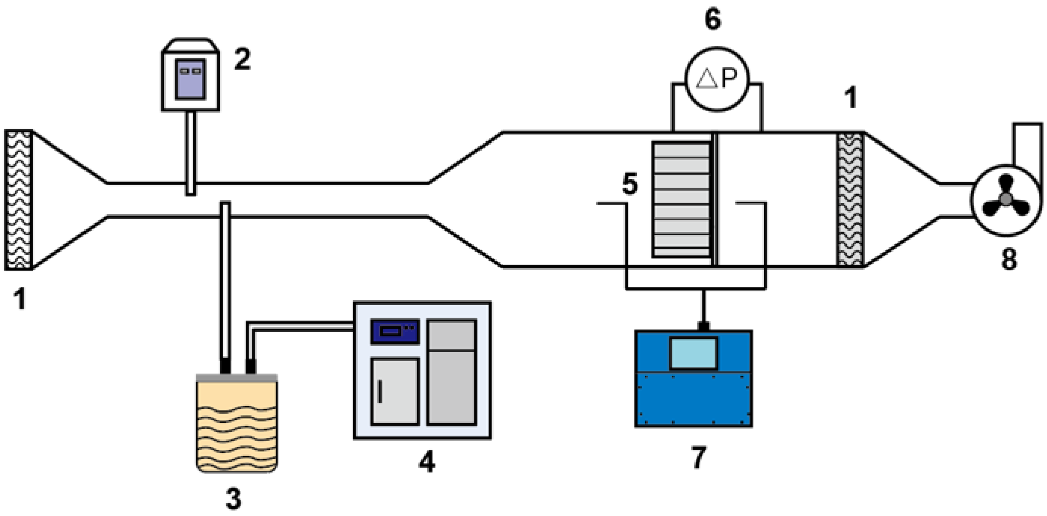

Figure S7. Schematic diagram of oil mist filter performance test device. 1 - High-efficiency air filter; 2 - Flow meter; 3 - Oil mist generator; 4 - Air compressor; 5 - Filter under test; 6 - Differential pressure gauge; 7 - Photometer; 8 - Vacuum fan.

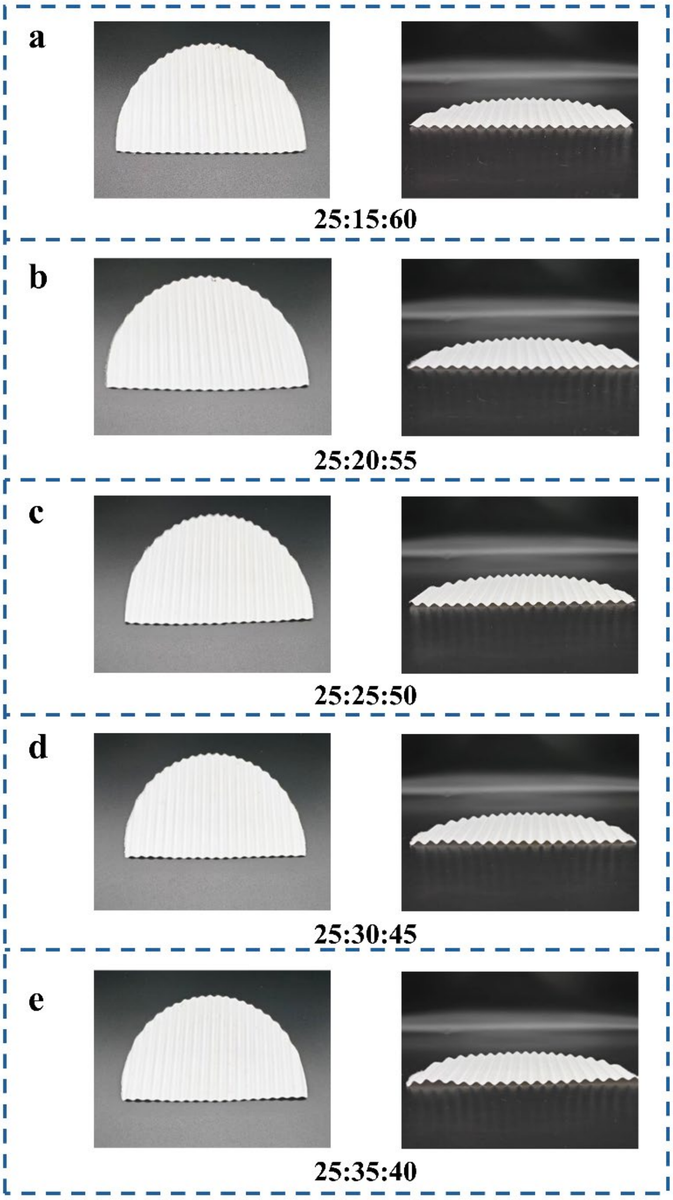

Figure S8. Photographs of corrugated-pleat-processed glass wool fiber/Tencel fiber blends with different blending ratios. (glass wool fiber 79 : glass wool fiber 59 : Tencel fiber, by ratio).

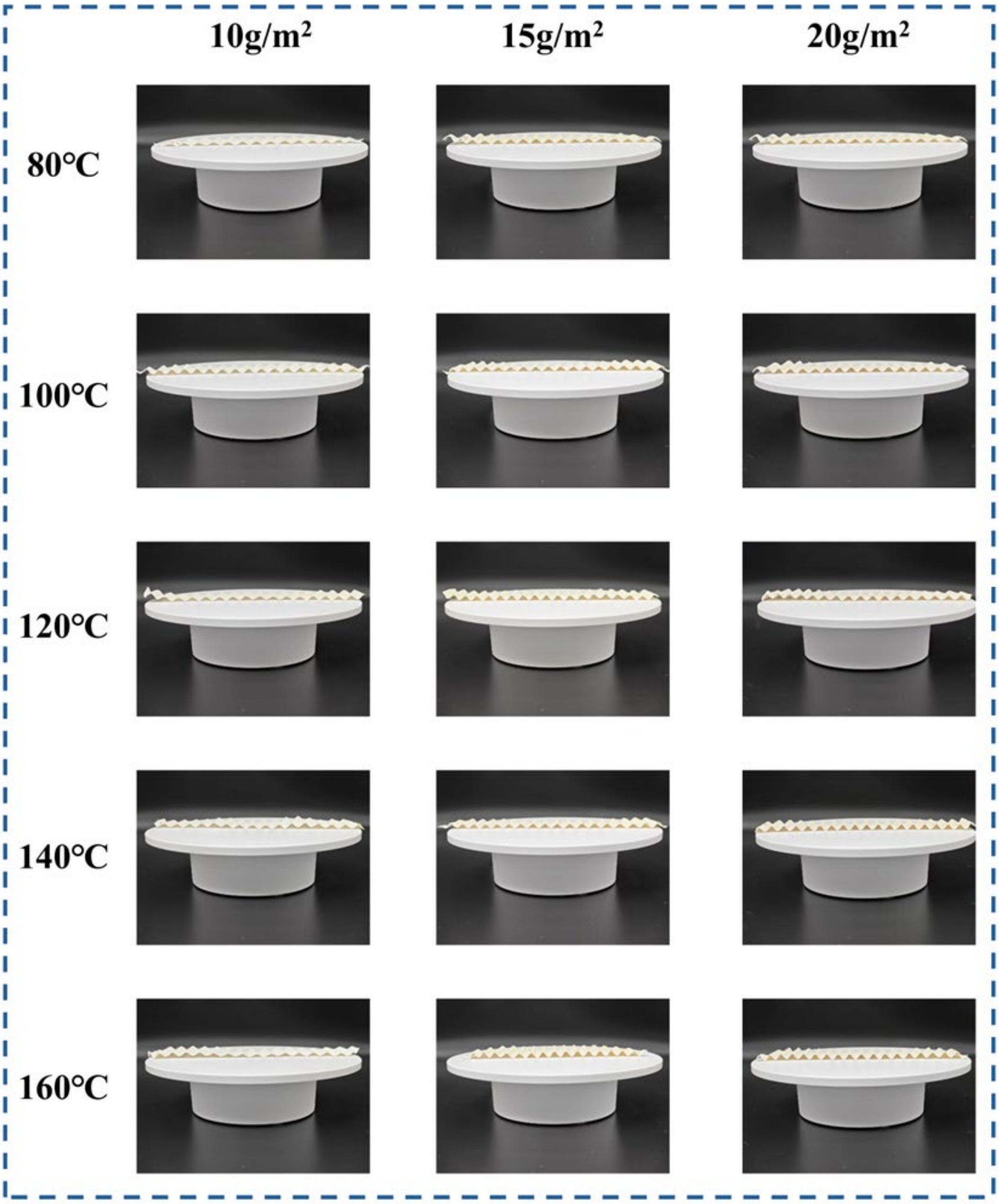

Figure S9. Photographs of composite filter media with three basis weight surface layers after pleating processing at different temperatures.
